# Extraction, Characterization, Biological Properties, and X-Ray Fluorescence Analysis of Functional Polysaccharides Derived from *Limnospira platensis*

**DOI:** 10.3390/life15081213

**Published:** 2025-07-31

**Authors:** Wanida Pan-utai, Naraporn Phomkaivon, Sarn Settachaimongkon, Preeyanut Pongponpai, Chomphunuch Songsiriritthigul

**Affiliations:** 1 Department of Applied Microbiology, Institute of Food Research and Product Development, Kasetsart University, Bangkok 10900, Thailand; 2 Department of Food Chemistry and Physics, Institute of Food Research and Product Development, Kasetsart University, Bangkok 10900, Thailand; ifrnpph@ku.ac.th; 3 Department of Food Technology, Faculty of Science, Chulalongkorn University, Pathumwan, Bangkok 10330, Thailand; sarn.s@chula.ac.th; 4 Faculty of Science, Prince of Songkla University, Songkhla 90110, Thailand; 5 Synchrotron Light Research Institute (Public Organization), 111 University Avenue, Nakhon Ratchasima 30000, Thailand; schomphunuch@gmail.com; 6 Center for Biomolecular Structure, Function and Application, Suranaree University of Technology, Nakhon Ratchasima 30000, Thailand

**Keywords:** microalga, *Spirulina*, functional, polysaccharide, X-ray fluorescence, biological, probiotic

## Abstract

This study explored the extraction, characterization, and biological properties of polysaccharides derived from *Spirulina* (*Limnospira platensis*), a microalga known for its rich nutritional benefits. Polysaccharides were successfully isolated and characterized using optimized biorefinery water extraction techniques to detail their structural and functional characteristics. Results revealed notable antioxidant activity and effective α-glucosidase inhibition, indicating potential health benefits. X-ray fluorescence (XRF) analysis was conducted to assess the elemental composition, offering insights into the mineral contents of the polysaccharides. Our findings underscore the promising applications of polysaccharides from *Limnospira platensis* as functional ingredients in health-related fields, advocating the need for further research into their mechanisms of action and therapeutic applications.

## 1. Introduction

*Spirulina*, classified under the genera *Arthrospira* or *Limnospira*, is a significant microalga renowned for its abundant nutritional content and diverse bioactive compounds [1]. This rich composition positions it as an important functional food, a valuable component in animal feed, and a promising resource in medicinal applications [2]. The diverse applications and nutritional profile of *Limnospira platensis* underscore its significance as a valuable resource. *Limnospira* is a blue-green microalga with a high growth rate, recognized for its significant polysaccharide content, contributing to its numerous health benefits. High-molecular-weight polysaccharides have demonstrated immunomodulatory, anti-inflammatory, and anticancer effects, as well as anti-aging properties [2,3,4]. *Limnospira* has a higher protein content than soybeans (55–70%) [5] and provides essential amino acids for the human body [6], with a carbohydrate content of 15–25% [7] and lipid content of 6–9% featuring essential fatty acids [6,8]. *Limnospira* also contains various pigments, minerals, vitamins, and phenolic compounds [6,9], making it a promising subject for exploration in health and wellness contexts, and polysaccharides derived from carbohydrates with glucose as a key component [10].

The polysaccharide composition of *Limnospira* is complex, consisting of various monosaccharides including rhamnose, mannose, xylose, and galactose, as well as two less common sugars [11]. These polysaccharides are critical for the biological functions of *Limnospira* and are currently undergoing investigation for their potential applications in health and medicine [12]. Polysaccharides from natural sources are increasingly recognized for their various beneficial biological activities for health applications. These macromolecules exhibit physiological effects such as antioxidant, anti-inflammatory, immunomodulatory, antitumor, antidiabetic, and prebiotic activities, making them promising candidates for functional foods and dietary supplements [12]. Polysaccharides from *L. platensis* (*Spirulina*) enhance the immune function, modulate gut microbiota, and improve insulin sensitivity. They also stimulate beneficial gut bacteria, regulate inflammation, and improve lipid and glucose metabolism, supporting their use in prebiotic formulations [10]. However, the link between the structural characteristics and functions of polysaccharides remains inadequately understood, with further research into their chemical properties and mechanisms required to facilitate integration into evidence-based therapeutic and nutritional applications. The polysaccharides in *Limnospira* provide an essential nutritional source for beneficial gut bacteria such as *Lactobacilli* and *Bifidobacteria* [10]. These microorganisms play a pivotal role in maintaining a healthy gut microbiome, thereby contributing to gastrointestinal health and well-being [13]. The extraction of polysaccharides from microalgae is a fundamental procedure for their isolation and subsequent applications. Various methodologies are available for polysaccharide extraction including water extraction, alkali extraction [14], enzyme extraction [12], and ultrasound-assisted methods [15]. Each of these techniques possesses distinct advantages and limitations, and the selection of a particular method can significantly influence the yield of the extracted polysaccharides. Careful selection of appropriate safety solvents is paramount during the extraction process, particularly when considering the bioactivity of the resultant polysaccharides [16]. Therefore, it is imperative to evaluate the effectiveness and safety of each extraction technique in the context of the intended applications of the functional polysaccharides.

Polysaccharides extracted from *Limnospira* have attracted significant interest due to their various biological properties and potential health benefits. The characterization of these polysaccharides is crucial for a comprehensive understanding of their structural attributes and functionalities [17]. This study investigated the extraction methods to characterize the biological properties associated with polysaccharides derived from *Limnospira*. Our findings underscore the importance of these compounds in wide ranging applications including food, pharmaceuticals, and nutraceuticals. In contemporary agricultural and multidisciplinary science, the analysis of trace elements in food and feed is increasingly conducted using synchrotron X-ray fluorescence (XRF). This technique has garnered significant interest by providing detailed elemental composition with high sensitivity and spatial resolution. Synchrotron XRF facilitates a deeper understanding of nutrient content and contamination levels in agricultural products, ultimately contributing to enhanced food safety and optimized agricultural practices. Research in this field continues to advance, and XRF technology presents promising opportunities for improving the quality and safety of food and feed [18,19,20,21,22,23].

This study investigated the efficacy of various extraction techniques on the yield and biochemical composition of polysaccharides extracted from *Spirulina* (*Limnospira platensis*). The antioxidant, α-glucosidase inhibition, and probiotic properties of polysaccharides were also evaluated to determine their potential as functional ingredients in health applications.

## 2. Materials and Methods

### 2.1. Microalgal Preparation and Composition

*Limnospira platensis* (formerly *Arthrospira platensis* or *Spirulina platensis*) IFRPD 1182 was obtained from the Institute of Food Research and Product Development (IFRPD), Kasetsart University, Thailand. *L. platensis* biomass preparation was carried out in Zarrouk medium, placed in a glass photobioreactor, and incubated in chamber equipment. The temperature was controlled at 30 °C with light intensity 162 µmol/m^2^/s using 18 W fluorescent daylight lamps and a 16/8 h light/dark cycle. Air mixed with 2% (*v*/*v*) CO_2_ was introduced at a flow rate of 0.67 vvm, passing through continuous bubbling via a PTFE membrane filter. The inoculum was prepared for 7 to 14 days or until the cells reached the logarithmic phase, and then utilized at 10% (*v*/*v*) to cultivate *L. platensis* biomass in 500 L raceway ponds filled with Zarrouk medium at a working volume of 200 L. A paddle wheel was adjusted to a speed of 15 rpm. During batch cultivation in open ponds, the average light photon flux density was 471 µmol/m^2^/s. The *L. platensis* biomass progressed to the log phase over 15 to 20 days, achieving a concentration of 1 g/L. A nylon membrane filtration was used for biomass harvesting, followed by washing with clean tap water until no culture medium residue remained. The collected cell biomass was dried in a hot air oven (UT6760; Thermo Scientific Heraeus Heating and Drying Ovens, Thermo Fisher Scientific Inc., Thermo Scientific, Dreieich, Germany) at 65 °C for 4 to 6 h until it contained less than 10% moisture. The *L. platensis* biomass was then ground to a particle size of 0.5 mm using a mill grinder (ZM-1, Retsch, Haan, Germany). Finally, the oven-dried *L. platensis* biomass was stored in aluminum foil packaging at room temperature before use.

The oven-dried *L. platensis* biomass was assessed for ash, protein, lipid, and crude fiber content following the AOAC standards [24]. The ash content was determined by burning dried samples in an electric furnace at 550 °C for 10 h. The protein content was measured using the Kjeldahl method, while the lipid content was extracted and analyzed via Soxhlet extraction with petroleum ether. Crude fiber was assessed using acid and alkaline techniques, with the resulting fiber residue dried until a stable weight was reached. The carbohydrate content was evaluated through the calculation of the difference between 100 g of dry matter and the cumulative values of moisture, protein, lipid, fiber, and ash contents.

### 2.2. Polysaccharide Extraction Procedures

Polysaccharide extraction from oven-dried *L. platensis* biomass was optimized using a two-level full factorial design that evaluated four critical factors to maximize polysaccharide concentration and extraction yield. These factors included biomass-to-solvent ratios of 1:20 and 1:40 (*m*/*v*), temperatures of extraction at 70 and 90 °C, extraction durations of 1 and 2 h, and 1 and 2 extraction cycles. The oven-dried biomass of *L. platensis* was combined with deionized distilled water and the extraction temperature was carefully controlled using a water bath (WNB10, Memmert, Schwabach, Germany). The combination was manually stirred every 10 min, and the resulting polysaccharides were isolated by centrifugation at 3660× *g* for 20 min (Frontier^TM^ 2000 Multi Centrifuges, Ohaus, Parsippany, NJ, USA). For the second extraction cycle, deionized distilled water was added to the biomass pellet residue, and the extracted polysaccharides from *L. platensis* were then stored at −20 °C for further analysis.

### 2.3. Determination of Polysaccharides Derived from L. platensis

#### 2.3.1. Polysaccharides

The polysaccharide concentration (PS) was evaluated using the phenol–sulfuric method, as described by Wang et al. [25] with minor modifications. First, 0.5 mL of polysaccharides extracted from *L. platensis* was placed in a test tube. Then, 5% phenol was added, and the mixture was blended using a vortex mixer. Next, 2.5 mL of sulfuric acid was incorporated, and the mixture was incubated at room temperature for 20 min. Finally, the samples were analyzed at 490 nm using a spectrophotometer (SP-8001, UV-Vis Spectrophotometer, Metertech, Taipei, Taiwan), with glucose as the standard reference. The polysaccharide content was calculated and expressed as glucose in mg/mL. The polysaccharide extraction yield was determined using Equation (1) and expressed in milligrams per gram (mg/g) biomass on a dry basis.

(1) $$Yield\left(\frac{mg}{g}\right)=\frac{PS\left(\frac{mg}{mL}\right)\times V\;(mL)}{M\;(g\;dry\;basis)}$$

where PS is the concentration of polysaccharides extracted (mg/mL), V is the volume of deionized distilled water used for extraction (mL), and M is the oven-dried *L. platensis* biomass dry basis (g).

#### 2.3.2. Total Phenolic Content

The total phenolic content (TPC) was assessed using the Folin–Ciocalteu method [26]. In summary, the standard or sample of 20 µL was mixed with 100 µL of 10% Folin–Ciocalteu reagent in a 96-well plate and incubated in the dark at room temperature for 8 min. Following this, 7.5% sodium carbonate and 50 µL of distilled water were added, and the mixture was stirred before being incubated at 40 °C for 30 min. The absorbance was then measured at 750 nm with a microplate reader (M965+, Microplate Reader, Metertech, Taiwan), using gallic acid as the standard. The TPC was presented as milligrams of gallic acid equivalent per gram of dry *L. platensis* biomass (mg GAE/g).

#### 2.3.3. Total Flavonoid Content

The total flavonoid content (TFC) was assessed utilizing a modified method [27]. A 100 µL aliquot of the standard or samples was combined with 100 µL of aluminum chloride solution. This mixture was incubated at room temperature for 10 min, after which the absorbance was measured at 405 nm (M965+, Microplate Reader, Metertech, Taiwan). Quercetin was used as the standard. The TFC was presented as milligrams of quercetin equivalent per gram of dry *L. platensis* biomass (mg QE/g).

#### 2.3.4. Reducing Sugars

Reducing sugars (R. sugars) were analyzed using the dinitrosalicylic acid (DNS) method, following the protocol established by Miller [28] with some modifications. A 0.25 mL standard or sample was combined with deionized water 0.5 mL and DNS reagent 0.25 mL. The mixture then underwent boiling in a water bath for 15 min, followed by cooling in cold water to terminate the reaction. Deionized distilled water (2 mL) was then added and the absorbance of the resulting solution was measured at 540 nm using a spectrophotometer (SP-8001, UV-Vis Spectrophotometer, Metertech, Taiwan). Glucose served as the reference for measuring reducing sugars, with the findings reported as glucose levels in milligrams per gram of dry *L. platensis* biomass (mg/g).

#### 2.3.5. Antioxidant Properties Using the DPPH Assay

The DPPH radical scavenging activity assay was conducted on polysaccharide-extracted samples derived from the biomass of *L. platensis*, following minor modifications [29]. The sample or standard (100 µL) was mixed with 100 µL of 200 µM DPPH solution (2,2-diphenyl-1-picrylhydrazyl, Sigma-Aldrich, Singapore). The mixture was kept in the dark and incubated for 30 min at room temperature. The absorbance was recorded at 750 nm with a microplate reader (M965+, Microplate Reader, Metertech, Taiwan). Ascorbic acid served as the reference standard. The antioxidant capacity was represented as milligrams of ascorbic acid equivalent per gram of dry *L. platensis* biomass (mg AAE/g).

#### 2.3.6. Antioxidant Properties Using the ABTS Assay

The ABTS radical scavenging activity assay was conducted on polysaccharide-extracted samples derived from the biomass of *L. platensis*, following a previously established method [26]. Briefly, the ABTS radical solution was formulated by combining 505.05 µL of 7 mM ABTS (2,2-azino-bis (3-ethylbenzothiazoline-6-sulfonic acid) diammonium salt (SRL, Mumbai, India) with 5.05 µL of 245 mM ammonium persulfate. The combination was left in a dark environment at room temperature for 16 h. The solution was then diluted with distilled water to attain an optical density of 0.7 at 750 nm. Next, 10 µL of the sample was combined with 190 µL of ABTS solution and the resultant mixture was stored in the dark for 5 min before the absorbance was measured at 750 nm utilizing a microplate reader. Ascorbic acid (Sigma-Aldrich, Singapore) was used as the standard reference. The antioxidant capacity was reported as milligrams of acid equivalent per gram of dry *L. platensis* biomass (mg AAE/g).

#### 2.3.7. Antioxidant Properties Using the FRAP Assay

The FRAP assay was used to measure the antioxidant capacity by assessing the ability of a substance to reduce ferric ions in the polysaccharide-extracted samples obtained from the biomass of *L. platensis*, following the established methodology [26]. The reagent was synthesized by mixing 300 mM sodium acetate at pH 3.6 with 10 mM TPTZ (2,4,6-tris (2-pyridyl)-s-triazine) (SRL, India). These components were dissolved in 40 mM HCl and mixed with 20 mM ferric chloride from Sigma-Aldrich, Singapore, using volumes of 25 mL, 2.5 mL, and 2.5 mL, respectively. Subsequently, the standard or 10 μL samples were combined with 190 µL of FRAP reagent. The mixtures were incubated in the dark for 30 min, and the absorbance was recorded at 593 nm with a microplate reader, using ascorbic acid (Sigma-Aldrich, Singapore) as the standard. The results were reported as milligrams of ascorbic acid equivalent per gram of dry *L. platensis* biomass (mg AAE/g).

### 2.4. Statistical Analysis

Parameters for polysaccharides derived from the microalga *L. platensis* were reported as mean values and standard deviations (SDs) for each set of three experiments. Statistical analyses were conducted using SPSS (SPSS, Inc., Version 25.0, Armonk, NY, USA). All the experimental parameters were assessed by Duncan’s multiple range test (DMRT) at a 0.05 significance level.

All parameter values were normalized and analyzed using the online platform Metaboanalyst 6.0 (www.metaboanalyst.ca, accessed on 23 November 2024) to facilitate the comparison of chemical profiles among the samples. Heatmap visualization, together with Pearson’s correlation-based hierarchical cluster analysis (HCA) and partial least squares discriminant analysis (PLS-DA), was employed to discern distinct patterns in the chemical profiles of the samples. Chemical parameters that displayed variable importance in projection (VIP) scores exceeding 1.0 and a *p*-value of ≤0.05 were identified as significant contributors to the differentiation of the samples. Pearson’s correlation analysis was performed on the chemical parameters and displayed using a hierarchically clustered correlation matrix and correlation pattern plots.

### 2.5. Polysaccharide Characterization and Properties

#### 2.5.1. Polysaccharide Preparation

Polysaccharides were extracted from *L. platensis* under the previously optimized condition [27]. The polysaccharide was prepared using a biomass-to-water ratio of 1:20 (*w*/*v*). Extraction temperature was controlled at 90 °C for 2 h. The extracted polysaccharides were centrifuged at 3660× *g* for 20 min to facilitate separation. Following this procedure, the samples were stored in a freezer at −20 °C for 18 to 24 h, and then freeze-dried at a pressure not exceeding 60 Pa for 40 h. The dry polysaccharides derived from *L. platensis* were processed to achieve a homogeneous particle size. The resulting freeze-dried polysaccharide powder was stored in aluminum foil packaging and maintained at −20 °C for future analysis and experimentation.

#### 2.5.2. Determination of Monosaccharides and Oligosaccharides

High-performance anion exchange chromatography (HPAEC) and a pulsed amperometric detector (PAD) were employed to analyze the freeze-dried polysaccharide powder derived from *L. platensis*. The chromatography system of ICS 5000 (Dionex™, Thermo Scientific, Vacaville, CA, USA) was fitted with a PA20 column (CarboPac™, 6 µm particle size, dimensions 3 × 150 mm) and a PA20 guard (CarboPac™, 6 µm particle size, dimensions 3 × 30 mm) and maintained at 30 °C. A “standard carbohydrate quad” waveform was utilized for signal detection, and data were processed using Chromeleon™ 6.8 Chromatography Data System software.

The procedure for releasing monosaccharides from the freeze-dried polysaccharide powder was adapted from methodologies outlined by Cai et al. [30]. A 2 mg/mL solution of freeze-dried polysaccharide powder was combined with 0.2 N trifluoroacetic acid (TFA) in a 1:1 (*v*/*v*) ratio and incubated in a dry bath (Boekel Scientific, Feasterville, PA, USA) at 100 °C for 2 h. Following evaporation, the acid-hydrolyzed polysaccharide powder was re-dissolved in deionized distilled water. Samples of the freeze-dried polysaccharide powder and the acid-hydrolyzed polysaccharide were injected at 10 µL into the column. The separation of monosaccharides was conducted using a modified approach based on Weitzhandler et al. [31], employing 16 mM sodium hydroxide in isocratic elution at a flow rate of 0.4 mL/min for 20 min.

The methodologies to determine oligosaccharides were adapted from the established protocols of Alyassin et al. [32]. A 10 µL aliquot of 1 mg/mL polysaccharide solution was injected into the chromatographic column. Gradient elution was conducted utilizing mobile phase A comprising 20 mM sodium hydroxide and mobile phase B consisting of 100 mM sodium hydroxide in conjunction with 250 mM sodium acetate. The elution profile was initiated at a composition of 100% A at 0 min, transitioning to 30% B at 18 min, then to 40% B at 25 min, ultimately achieving 100% B by 27 min. A concentration of 200 mM sodium hydroxide was maintained for 4 min before equilibrating with 100% A for 15 min, thus preparing for subsequent injections.

A calibration curve for monosaccharides and oligosaccharides was developed using a series of dilutions of eleven defined standards: arabinose, galactose, glucose, fructose, rhamnose, xylose, maltose, sucrose, isomaltose, panose, and maltotriose (Sigma-Aldrich, Singapore). These standards were systematically diluted to ensure accurate quantification and validation of the analytical methods employed throughout this study.

#### 2.5.3. X-Ray Fluorescence (XRF) Analysis

Here, 60 mL of *L. platensis* biomass powder and polysaccharide powder extracted from *L. platensis* was used to make a cylindrical pellet 10 mm in diameter and 0.4 mm in thickness by a press machine (Specac, Orpington, UK) for 1 min at a force of 1 ton. The pellets were adhered to Kapton tape (Lanmar Inc., Northbrook, IL, USA) in a Supralene frame and placed in an acrylic sample chamber for X-ray exposure.

**Figure 1:**
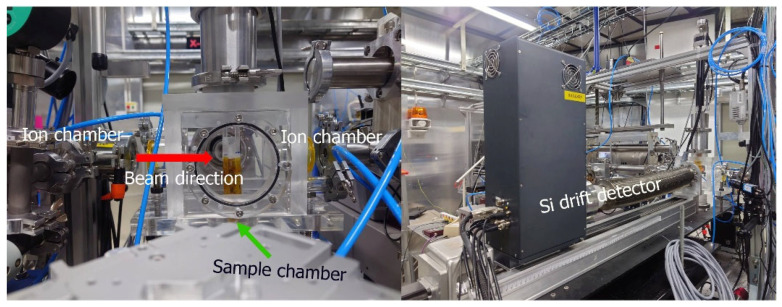
The X-ray fluorescence (XRF) experimental setup on Beamline 7.2 W comprising ion chambers, the sample chamber, and an Si drift detector.

XRF measurement was performed on the BL7.2W beamline of the Synchrotron Light Research Institute (SLRI), Thailand. A monochromatic focused X-ray beam with a size of 4 × 2 mm^2^ (hor. × ver.) was exposed to a sample placed at 45 degrees to the incident X-ray beam. A four-element silicon drift detector (SDD), Vortex@-ME4 (Hitachi High-Tech Science America, Inc., Chatsworth, CA, USA), with a 10 mm sleeve diameter, was positioned perpendicular to the sample to capture the emitted fluorescent X-rays. Measurement of X-ray fluorescence was performed with an X-ray energy of 12.90 keV and an exposure time of 20 min for sample measurement in a helium atmosphere. Helium gas was purged in the acrylic sample chamber to ensure high efficiency in detecting low-mass elements such as S, Cl, K, and Ca. The energy of the incident beam tuned by a Si (111) double-crystal monochromator was chosen depending on the elements of interest, and all the measurements were conducted in triplicate. The sample-to-detector distance was 7.5 cm. The photon flux of 12.9 keV monochromatic X-rays with a double Si (111) crystal measured by an ion chamber was 1.05 × 10^11^ photons/s at 100 mA stored electron beam. Beamline specifications and performance were previously reported [33]. Figure 1 shows a picture of the experimental setup and the apparatus. The sample was placed in the sample chamber and exposed to a monochromatic-focused X-ray beam. The XRF spectrum was analyzed via PyMCA (version 5.5.5) [34], which was created by the Software Group at the European Synchrotron Radiation Facility (ESRF) (http://pymca.sourceforge.net/PyMca).

To assess the performance of the measurement system, the limit of quantification (LOQ) was established using the certified reference material of lichen (CRM 482) [35,36], thereby assuming a similar matrix to that of the analyte samples. Subsequently, the presence of elements in these two samples was determined based on the limit of quantification (LOQ) specific to the experimental geometry. The LOQ was quantified with a percentage of relative standard deviation (%RSD) not exceeding 10% [37]. The LOQ calculated from CRM 482 for chromium, nickel, copper, zinc, and arsenic were 1.76 ppm, 0.33 ppm, 0.29 ppm, 0.13 ppm, and 0.11 ppm, respectively.

Without the need for calibration standards, the “fundamental parameters” (FP) method was executed in PyMca, thus accounting for matrix effects and calibrating against standard reference material. To leverage the advantages of the FP method for XRF quantification, the geometrical arrangement of the source, sample, and detector was optimized and maintained consistently throughout the experiment. The estimation of analytical accuracy, expressed as the percentage difference from the certified value as determined by the FP method, was performed. Using a 95% confidence interval (2σ error values, 20% relative error of the calculated value), the calculated mass fractions of the elements reported in CRM 482 (Cr, Ni, Cu, Zn, and As) were within ±22% of the certified values. Subsequently, the configuration file for the FP method in PyMca, which corresponds to the mass fractions of specific elements in CRM 482, was further employed for sample analysis.

#### 2.5.4. The Effects of Probiotic Proliferation

The impact of freeze-dried polysaccharide powder derived from *L. platensis* on the growth of probiotics was refined using a more precise methodology [38], with *Lactobacillus rhamnosus* ATCC 53103 obtained from the American Type Culture Collection (ATCC, Manassas, VA, USA) and *Bifidobacterium longum* subsp. *longum* TISTR 2195 obtained from the Thailand Institute of Scientific and Technological Research (TISTR, Pathum Thani, Thailand) utilized as reference strains. *L. rhamnosus* ATCC 53103 and *B. longum* TISTR 2195 were cultured in MRS and MRS supplemented with 0.05% L-cysteine, respectively. *L. rhamnosus* was incubated at 37 °C for 18 h. *B. longum* was incubated under anaerobic conditions using an AnaeroPack (AnaeroPack-Anaero, Mitsubishi Gas Chemical Company, Inc., Tokyo, Japan) at the same temperature for 18 h. Following this, 10% (*v*/*v*) of the culture was transferred into fresh medium broth and incubated at the same temperature for an additional 12 h. The cells were then centrifuged at 3660× *g* for 10 min, subjected to two wash cycles with 0.1% peptone, and resuspended in a 0.1% peptone solution. The probiotic bacterial preparation, prepared by diluting the cell suspension, achieved an absorbance of 0.2 at an optical density of 600 nm. Freeze-dried powder derived from *L. platensis* at 2% (*w*/*v*) was supplemented in the minimum medium broth. The control utilized glucose and inulin as reference standards. The suspension preparations of *L. rhamnosus* ATCC 53103 and *B. longum* TISTR 2195, at 10% (*v*/*v*), were added to the test and were incubated at 37 °C for 48 h. The viable cells of *L. rhamnosus* ATCC 53103 and *B. longum* TISTR 2195 were quantified using the plate count method on MRS agar and MRS agar supplemented with 0.05% L-cysteine, respectively, during cultivation for 48 h.

#### 2.5.5. Inhibition of α-Glucosidase

The sample preparation involved mixing 50 mg of oven-dried *L. platensis* biomass with 1 mL of deionized distilled water and allowing the mixture to rest for 5 min before centrifuging at 3660× *g* for 5 min. Freeze-dried polysaccharide powder (2 mg) derived from *L. platensis* was dissolved in 1 mL of deionized distilled water. The supernatant was collected and kept at −20 °C for future analysis.

The α-glucosidase activity of oven-dried *L. platensis* biomass and freeze-dried polysaccharide powder was evaluated using established methodologies [39,40]. A 50 µL aliquot of each sample solution, with concentrations ranging from 1 to 50 mg/mL, was placed in a 96-well plate. Then, 100 µL of α-glucosidase solution (1 U/mL) in a 0.1 M phosphate buffer (pH 6.9) was added, and the mixture was incubated at 37 °C for 10 min. Next, 50 µL of *p*-nitrophenyl-α-D-glucopyranoside solution (5 mM in buffer) was introduced into each well. The mixture was incubated again at 37 °C for 5 min and the absorbance of the reaction was measured using a microplate reader (Tecan Infinite 200 PRO, Tecan Inc., Grödig, Austria) at a wavelength of 405 nm. The inhibition of α-glucosidase activity was calculated using the following equation

(2) $$Inhibition\;\left(\%\right)=\frac{A_{control}-A_{sample}}{A_{control}}\times 100$$

where $A_{control}$ is the absorbance of the control using the buffer instead of the sample and $A_{sample}$ is the absorbance of the sample. The sample concentration that inhibited α-glucosidase activity by 50% (IC_50_ value) was calculated.

## 3. Results

Polysaccharides extracted from the oven-dried biomass of *Limnospira platensis* utilizing a biorefinery water extraction method were characterized by various analytical methods to elucidate their structural and functional properties and their impact on probiotic growth.

### 3.1. Biochemical Composition

The microalgal biomass of *Limnospira platensis* was oven-dried to determine the biochemical composition. Table 1 presents the proximate composition of the oven-dried biomass of *Limnospira platensis*. The protein content was the most prominent component, highlighting the nutritional value of this microalga. Carbohydrates and crude fiber were 16.87% and 16.26% (% dry basis). Findings suggested that substantial amounts of carbohydrates were retained within the cells of *L. platensis*, reflecting the intricate biochemical processes that contributed to its unique composition.

### 3.2. Polysaccharide Extracts Derived from L. platensis

Polysaccharides were extracted from oven-dried biomass of *L. platensis* using water extraction under varied conditions. The polysaccharide concentration and extraction yield are shown in Table 2. The polysaccharide (PS) concentration and extraction yield ranged from 0.07 to 2.48 mg/mL and 1.56 to 54.62 mg/g, respectively. The highest polysaccharide concentration was obtained at a biomass/water ratio of 1:20 and an extraction duration of 2 h. By contrast, the highest extraction yield was achieved at a biomass/water ratio of 1:40 and an extraction time of 1 h. An extraction yield of 54.62 mg/g at 70 °C and 1 h was not significantly different from the yield at 90 °C for extraction times of 1 and 2 h.

Polysaccharide extracts obtained from *L. platensis* under various conditions were analyzed to determine the total phenolic, flavonoid, and reducing sugar contents in the supernatant (Table 3). The highest concentrations were achieved during the initial extraction cycle compared with subsequent extractions. The maximum concentrations of TPC and TFC were achieved at a biomass/water ratio of 1:40, with an extraction temperature of 90 °C, for an extract duration of 2 h. By contrast, a biomass/water ratio of 1:20 produced the maximum levels of reducing sugars compared with the other ratios. The TPC and TFC obtained from a biomass/water ratio of 1:40 at conditions of temperature 90 °C for 2 h during the initial extraction cycle were 3.66 mg GAE/g and 0.90 mg QE/g, respectively. By contrast, the highest recorded reducing sugar content of 4.77 mg/g was achieved with a biomass/water ratio at 1:20 with a temperature of 70 °C for one hour during the first extraction cycle.

The antioxidant properties of functional polysaccharides extracted from *L. platensis* under DPPH, ABTS, and FRAP assays are summarized in Table 4. The DPPH assay values ranged from 0.12 to 0.61 mg AAE/g, while the ABTS assay gave results between 0.482 and 2.27 mg AAE/g, and the FRAP assay yielded values from 0.46 to 1.91 mg AAE/g. The first extraction cycle exhibited superior antioxidant properties to the second cycle across all the experiments. The highest DPPH antioxidant activity was recorded with a biomass/water ratio of 1:20 at 90 °C for 1 h. By contrast, the optimal antioxidant properties for the ABTS and FRAP assays were achieved at a biomass/water ratio of 1:40, extracted for 1 h at temperatures of 70 °C and 90 °C, respectively.

### 3.3. Comparison of Chemical Profiles

**Figure 2:**
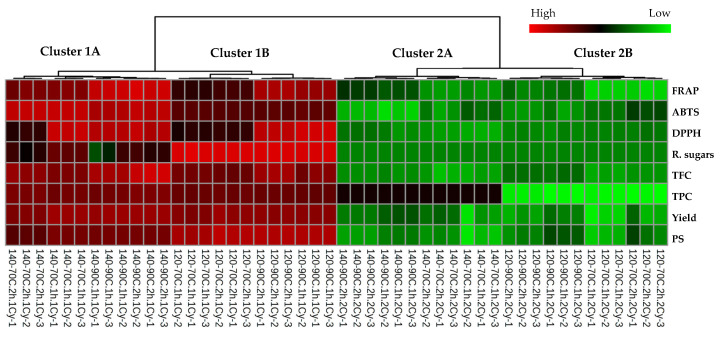
Heat map and hierarchical clustering of the chemical profiles of extracted polysaccharide samples from *L. platensis* under various conditions. The dendrogram illustrates the clustering of samples using average linkage based on Pearson’s correlation coefficients. Each square denotes the normalized chemical abundance, with red indicating a higher content of the respective chemical parameters. To facilitate the interpretation of the color references depicted in this figure, the reader is advised to consult the statistical comparisons of the chemical properties of the samples presented in Table 2, Table 3 and Table 4.

A non-supervised Pearson’s correlation-based hierarchical clustering combined with a heat map was performed to evaluate the similarities among the chemical profiles of polysaccharides extracted from *L. platensis* under various conditions (Figure 2). Results revealed distinct variations in the chemical profiles between samples from the initial and subsequent extraction cycles (cluster 1 vs. 2). The chemical profiles of samples from the same extraction cycle were significantly affected by changes in the biomass/solvent ratio (cluster A vs. B). A heat map applies color gradients to represent the relative abundance of chemical features across samples, with a red hue denoting higher value and a green hue indicating lower value of the observed chemical features. Color shading in the heat map indicated that samples from the first extraction cycle (cluster 1) exhibited higher levels across all the measured chemical parameters. Based on this finding, further chemometric analysis was restricted to the chemical profiles of samples derived from the initial extraction cycle to investigate the impact of the biomass/solvent ratio and extraction temperature and duration.

**Figure 3:**
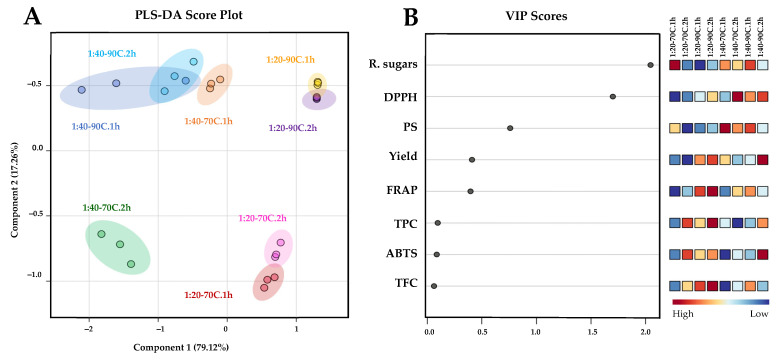
PLS-DA score plot (**A**) illustrating the chemical profile comparison of extracted polysaccharide samples from *L. platensis* under various conditions. Key features accountable for discrimination are ranked in descending order based on their variable importance in projection (VIP) scores (**B**). Squares in the VIP score panel represent normalized chemical abundance, with red hue denoting a higher content of the corresponding parameter. To facilitate the interpretation of the color references depicted in this figure, the reader is advised to consult the statistical comparisons of the chemical properties of the samples presented in Table 2, Table 3 and Table 4.

A comparison of the chemical profiles among the samples from the initial extraction cycle is presented in Figure 2. Partial least squares discriminant analysis (PLS-DA) was employed to distinguish the chemical profiles of samples obtained from the first extraction cycle. A score plot was constructed with a prediction accuracy of 75.54%, *R*^2^ = 0.738, and *Q*^2^ = 0.611 (Figure 3A). Results demonstrated clear distinctions in chemical profile patterns between the samples extracted at biomass/solvent ratios of 1:40 and 1:20 along component 1, accounting for 79.12% of the variance. Variations in the chemical profiles of the samples, attributable to extraction temperatures of 70 °C and 90 °C were captured along component 2 (17.26%). This result corresponded with the HCA-derived dendrogram pattern mentioned previously. PLS-DA-derived VIP scores with values greater than 1.0 (Figure 3B) and *p* < 0.05 (Table 1, Table 2 and Table 3) suggested that variations in reducing sugar, DPPH, and PS contents were indicative parameters for the differentiation of polysaccharides extracted from *L. platensis* under various conditions.

**Figure 4:**
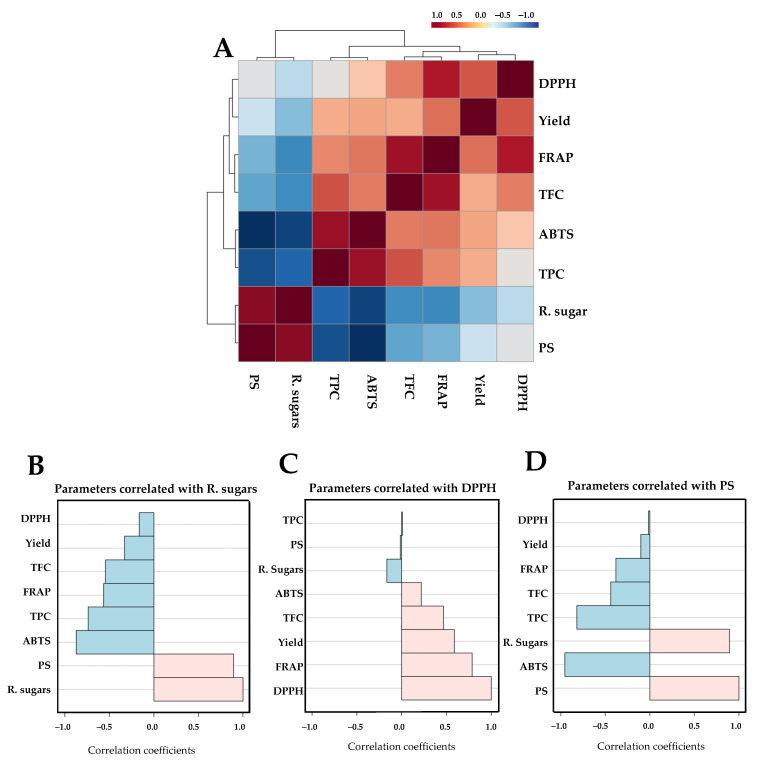
Hierarchical clustering correlogram of eight chemical parameters based on Pearson’s correlation analysis (**A**). Each colored cell represents the correlation coefficient between parameter pairs, with red and blue indicating positive and negative correlations, respectively. Panels (**B**–**D**) display correlation coefficient plots for three representative parameters, i.e., residual sugar (**B**), DPPH (**C**), and PS (**D**), visualized as horizontal bar graphs, where light pink indicates positive correlations and light blue indicates negative correlations.

The correlation analysis among the various chemical parameters is presented in Figure 3. A Pearson correlation coefficient matrix comprising eight chemical parameters was constructed as a correlogram (Figure 4A). The results indicated the formation of two distinct clusters, characterized by positive (red shading) and negative (blue shading) correlations among the measured parameters. Key chemical features, specifically reducing sugar, DPPH, and PS, with VIP scores greater than 1.0 were selected to examine their correlation coefficients with other parameters. Parameters were considered strongly associated when Pearson’s correlation coefficient was greater than 0.5. The reducing sugar content of the extracted samples showed a positive correlation with PS and a negative correlation with ABTS, TPC, FRAP, and TFC (Figure 4B), with the DPPH value positively correlated with FRAP and extraction yield (Figure 4C). The PS level showed a positive correlation with the amount of reducing sugar and a negative correlation with ABTS and TPC (Figure 4D).

### 3.4. Monosaccharide and Oligosaccharide Compositions

The polysaccharide powder derived from *L. platensis* was characterized by its monosaccharide and oligosaccharide composition. A comprehensive analysis enabled the identification and quantification of the various carbohydrate components, providing valuable insights into the biochemical properties and potential applications of the polysaccharides extracted from this microalga. The selected monosaccharides, including arabinose, galactose, glucose, fructose, rhamnose, and xylose, were measured using HPAEC-PAD (Table 5). Six monosaccharides were eluted for between 6.507 and 12.217 min under isocratic conditions. The polysaccharide powder derived from *L. platensis* contained low amounts of glucose and fructose (less than 10 mg/g). Three predominant monosaccharides (mg/g) in the *L. platensis* acid-hydrolyzed polysaccharide powders were glucose (63.27), rhamnose (21.99), and galactose (16.14), with minor compositions of xylose, arabinose, and fructose.

**Figure 5:**
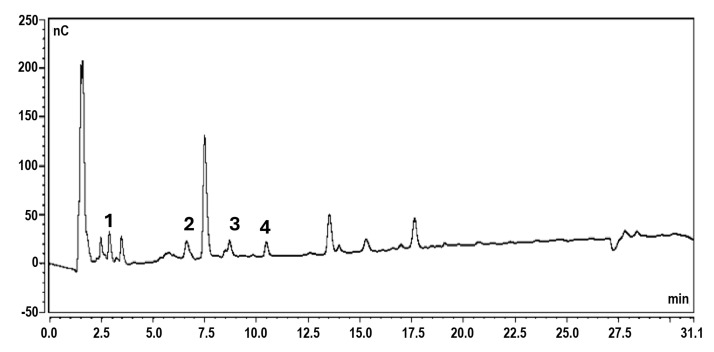
Chromatogram illustrating the oligosaccharide composition of polysaccharide powder derived from *L. platensis.* (1: glucose, 2: maltose, 3: panose, and 4: maltotriose).

A chromatogram illustrating the oligosaccharide composition of polysaccharide powder derived from *L. platensis* is presented in Figure 5. The peaks represent various oligosaccharide fractions, highlighting their distinct profiles and concentrations, which are critical for understanding the structural and functional properties of the biopolymer. Glucose (peak 1) was detected in the extract at 2.9 min, while maltose (peak 2) was separated at 6.6 min. The concentrations of mono- and disaccharides were measured at 9.67 mg/g and 35.27 mg/g, respectively. Trisaccharides appeared at 8.7 min (peak 3) and 10.5 min (peak 4), corresponding to panose and maltotriose. The predominant oligosaccharides in the spirulina extract were panose and maltotriose at 55.51 mg/g and 51.51 mg/g, respectively. The remaining peaks, ranging from 12.0 to 25.0 min, likely corresponded to short-chain oligosaccharides containing glucose molecules (gluco-oligosaccharides, GlcOSs). Glucose was the major polysaccharide component extracted from spirulina, with maltose, maltotriose, and panose also present. Maltose consists of two glucose molecules linked by an α-1,4-glycosidic bond, while maltotriose comprises three glucose molecules connected via an α-1,4-glycosidic bond. Panose is formed by a maltose unit linked to an additional glucose molecule through an α-1,6-glycosidic bond. The distinct structures of these GlcOSs play a crucial role in their biological activities, which include prebiotic potentials and health benefits.

### 3.5. Synchrotron-Based X-Ray Fluorescence

**Figure 6:**
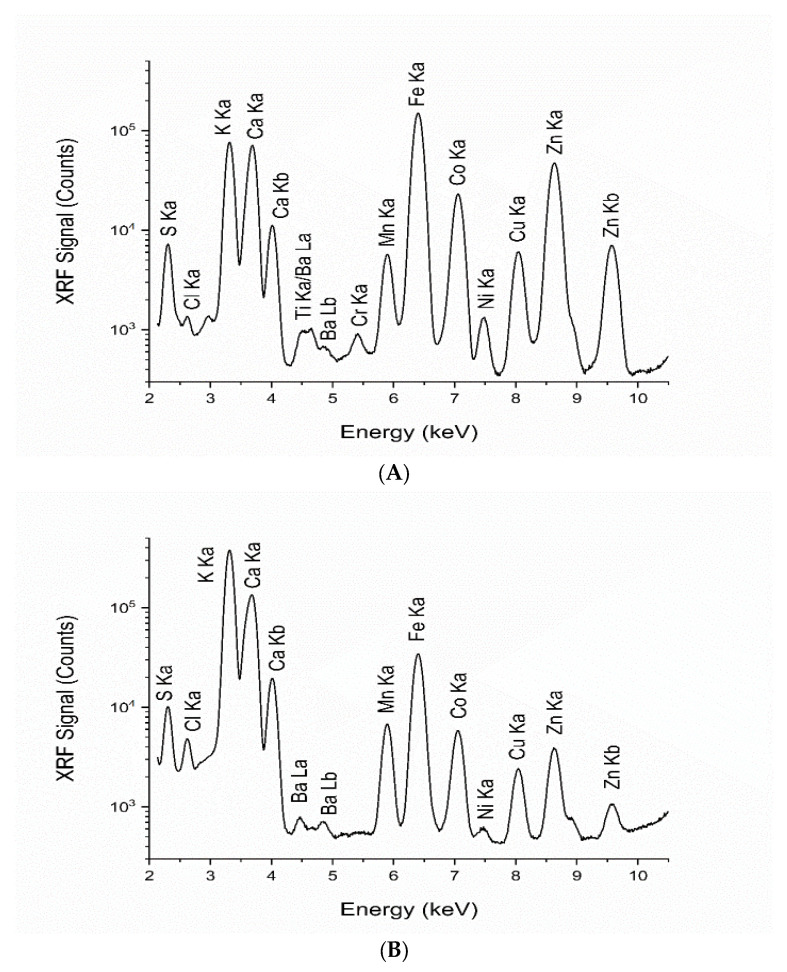
X-ray fluorescence spectra of (**A**) oven-dried biomass of *L. platensis* and (**B**) polysaccharide powder derived from *L. platensis*.

The X-ray fluorescence spectra of *L. platensis* oven-dried biomass and polysaccharide powder derived from *L. platensis* are shown in Figure 6A and Figure 6B, respectively. The XRF technique simultaneously detected various elements. Inorganic mineral nutrients (S, Cl, K, Ca, Mn, and Fe) were detected as the major and minor elements in the two samples of spirulina and the extract. The weight percentages of the elements contained in the samples using XRF are reported in Table 6. The RSD values of the two sample measurements were within the range 0.1% to 11% and acceptable for the elemental concentrations [37].

### 3.6. Inhibition of α-Glucosidase

**Figure 7:**
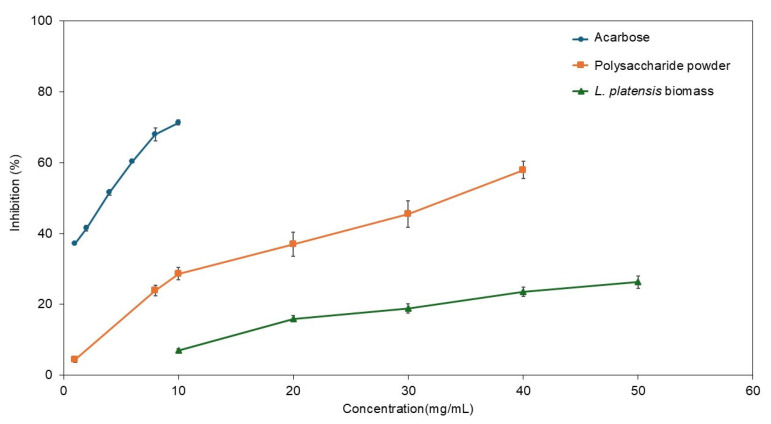
Comparative analyses of α-glucosidase inhibition activities among oven-dried *L. platensis* biomass, polysaccharide powder derived from *L. platensis*, and the standard inhibitor acarbose.

The inhibitory effects on α-glucosidase activity among oven-dried *L. platensis* biomass, polysaccharide powder derived from *L. platensis*, and the standard inhibitor acarbose are illustrated in Figure 7. The polysaccharide powder derived from *L. platensis* exhibited greater suppression of α-glucosidase activity than its initial oven-dried biomass raw material, doubling the effectiveness. The highest inhibitory effects recorded were 57.9% for the polysaccharide powder at a concentration of 40 mg/mL and 26.3% for the oven-dried *L. platensis* biomass at 50 mg/mL. The suppression of α-glucosidase activity increased at higher sample concentrations.

The α-glucosidase inhibition activities in biomass and polysaccharide powder derived from *L. platensis* were evaluated (Table 7). The IC_50_ values for oven-dried *L. platensis*, polysaccharide powder derived from *L. platensis*, and acarbose were >50 mg/mL, 33.01 mg/mL, and 3.41 mg/mL, respectively. The IC_50_ value of the polysaccharide powder was 9.7 times higher than acarbose. These findings suggest that polysaccharide extraction from *L. platensis* is an effective strategy to develop new sources of biological compounds, ensuring a safer process with reduced chemical use.

## 4. Discussion

*Limnospira platensis*, a blue-green alga, has a well-known nutritional composition and functional properties [41]. A previous paper recorded the carbohydrate content in *L. platensis* biomass to be 2.42 to 30.7% [42]. Carbohydrate accumulation in *Limnospira platensis* is influenced by multiple factors including the specific strain utilized, the cultivation methodology, the composition of the growth medium, and environmental variables such as light intensity, temperature, agitation rates, and carbon dioxide concentration [43,44,45]. In this study, the carbohydrate content of *L. platensis* was similar to previous reports at 10–14% [46]. Polysaccharide extraction from *L. platensis* was conducted utilizing water as a sustainable extraction method. The cell wall of *Limnospira* sp. is predominantly composed of intricate heteropolysaccharides and glycoproteins, indicating that *Limnospira* is void of cellulose, thereby rendering its cell wall more digestible and less rigid [47,48]. These polysaccharides are essential for maintaining cellular morphology, providing structural support and protecting the cell from environmental stressors [49]. Our results indicated that water-soluble polysaccharides from *L. platensis* were influenced by biomass concentration, extraction temperature and duration, and the number of extraction cycles. An increase in biomass concentration was associated with a higher level of polysaccharides. Enhancing the biomass/solvent ratio during polysaccharide extraction initially boosted the yield of polysaccharides [50], while increasing the extraction temperature and duration significantly enhanced polysaccharide yield during the extraction process. Elevated temperatures within an appropriate range improved the solubility of polysaccharides and facilitated their release into the extraction solvent. However, excessively high temperatures or extended extraction times promoted the degradation of polysaccharides, adversely affecting their yield and structural integrity [51]. Elevated temperatures enhanced the diffusion rates of polysaccharides, thereby facilitating their extraction from the biomass [52]. Temperatures above a critical threshold of 180 °C led to the degradation of proteins and also the thermal degradation of polysaccharides [53]. Extended extraction durations resulted in the breakdown of polysaccharides, ultimately decreasing the total yield [54]. Results revealed an increase in polysaccharide concentration at a high biomass/water concentration of 1:20% *w*/*v*, particularly when the extraction temperature was set at 7 °C, producing polysaccharide concentration results similar to those observed at 90 °C. By contrast, the extraction process gave the highest output at a lower biomass concentration of 1:40% *w*/*v*, demonstrating comparable results across various extraction temperatures and time durations and showcasing the nuanced impact of these parameters on extraction efficiency. Our findings suggest that repetition of the extraction process during the second cycle is ineffective for polysaccharide extraction from *L. platensis*, attributed to the absence of a cell wall, which only facilitates efficient extraction of polysaccharides in the initial cycle. Water extraction was selected as the primary method due to its alignment with green chemistry principles, cost-effectiveness, and safety for applications involving food, nutraceuticals, and probiotics. Unlike chemical (e.g., alkaline) or enzymatic methods, water extraction avoids the use of harsh reagents or costly enzymes, thereby preserving the native structure and functional integrity of bioactive compounds such as polysaccharides and pigments [55,56]. Moreover, water extraction minimizes residual contamination, making it particularly suitable for downstream biological or probiotic assays where purity and non-toxicity are critical [57]. Ultrasound-assisted extraction (UAE), enzyme-assisted extraction (EAE), or alkali treatment enhance extraction efficiency and yield but may also lead to degradation or structural modification of sensitive compounds [58,59]. These methods often require the optimization of multiple parameters (e.g., enzyme type/concentration, pH, temperature), which increases processing complexity and cost. This study prioritized a clean-label and scalable approach, suitable for functional food applications.

A previous report detailed the extraction conditions for polysaccharides from *Limnospira* using a range of methodologies. Each methodology has unique conditions that yield varying results. Conventional hot water extraction typically yields 8.35% polysaccharides from *L. platensis* at 80 °C for 8 h [60]. This extraction technique is notable for its efficacy in isolating valuable polysaccharide compounds, highlighting its relevance in the field of biopolymer research and applications. The controlled parameters of temperature and time play crucial roles in optimizing the yield and quality of the extracted polysaccharides, making this method popular within natural product chemistry [61]. Alkali extraction significantly enhanced the yield of polysaccharides from *L. platensis*, with 65% efficiency under optimal conditions. Key parameters for this process included biomass-to-solvent ratio 1:50 (% *w*/*v*), alkaline pH of 10.25, and extraction at a controlled temperature of 89.24 °C for 10 h. These conditions are crucial for maximizing polysaccharide extraction and demonstrate the effectiveness of alkali treatment in extracting valuable compounds from algal biomass [14]. Ultrasonic-assisted extraction techniques have demonstrated significant efficacy in enhancing polysaccharide yields from various sources. A recent study involving *L. platensis* reported a crude polysaccharide yield of 14.78% under optimized conditions utilizing a biomass-to-solvent ratio of 1:30% *w*/*v* at 81 °C for 30 min and an ultrasonic power setting of 92 W. These parameters indicate the potential of ultrasonic assistance in maximizing polysaccharide extraction efficiency from microalgal sources [62]. The optimal conditions for polysaccharide extraction from microalgae are influenced by multiple factors and methods, which must be analyzed and considered for their implications in subsequent applications.

The extraction procedure employed for *L. platensis* facilitated the isolation of polysaccharides and also various other bioactive compounds. Our investigation revealed that the water-soluble polysaccharides derived from *L. platensis* contained significant levels of total phenolic and flavonoid contents, and a notable quantity of reducing sugars with antioxidant properties. These findings underscored the potential of *L. platensis* as a source of bioactive substances with possible health benefits. A previous report indicated that increasing the extraction temperature led to higher phenolic content and enhanced antioxidant activity. Hot water extraction gave higher TPC, TFC, and antioxidant activity compared with extraction at room temperature [63], supporting the effectiveness of our polysaccharide extraction method. Exploring the diverse chemical profiles of polysaccharides obtained from *L. platensis* unveiled fascinating insights. The levels of reducing sugars, DPPH, and polysaccharide content emerged as pivotal indicators. These parameters showed the potential to characterize the variations in extracted polysaccharides under different conditions throughout this study, highlighting the intricate relationship between extraction methods and the biochemical composition of these compounds.

The extraction yield of polysaccharides from *L. platensis* obtained through hot water or mild method set up to 54.62 mg/g significantly exceeded other microalgae such as *Chlorella* sp. (13–19%) [64,65], and was higher than the yields achieved through ultrasonication for *Caulerpa lentillifera* (7.8%) [66]. The bioactive compounds—TPC (3.66 mg GAE/g) and TFC (0.90 mg QE/g)—were similar to extracts from *Haematococcus pluvialis* (around 4 mg GAE/g) and exceeded plant fiber extracts like inulin or pectin [67]. Water-based extraction has moderate efficiency but is a low-cost, safe, and food-grade method that is competitive with more complex techniques such as microwave or pressure-assisted extraction. Specialized methods may enhance yield and selectivity, but our extraction approach provides eco-friendly, sustainable, and food-grade results with minimal processing.

The polysaccharides derived from *Limnospira* were primarily characterized by their average molecular weight, the types of monosaccharides present, and the positioning of their glycosidic bonds. Results in Table 8 show that the most prevalent monosaccharide in *Limnospira* sp. from various locations was glucose, followed by rhamnose and galactose. The monosaccharide profile following acid hydrolysis of *L. platensis* polysaccharide extracts aligned with findings from previous studies. The accumulation of glucose within the cell body is related to the photosynthetic activity of cyanobacteria, which convert carbon dioxide into sugars [68]. By contrast, the production of other monosaccharides corresponds to the clumping behavior observed in cyanobacteria, particularly the significant amount of rhamnose [69]. Variations in monosaccharide composition result in unique functional properties and food applications such as thickening agents, stabilizers, and flocculants [68,70,71]. The presence of free monosaccharides in minimal amounts indicated that the sugar composition in spirulina is associated with polymer structures [7]. A previous report identified a prebiotic oligosaccharide (SPO-1 fraction) featuring glucose molecules with an α-1,4-glycosidic linkage sourced from spirulina [30]. To date, the biological activities attributed to *Limnospira* include antioxidant, anticancer, anti-inflammatory, and antidiabetic properties as well as prebiotic potentials [30,72,73].

The beneficial effects of oligosaccharides in *Limnospira*, particularly in the context of probiotic activity, are well-documented in contemporary research. Short-chain oligosaccharides (with a degree of polymerization ranging from 3 to 10) play a crucial role in promoting the growth of health-beneficial bacteria through selective fermentation and the production of advantageous metabolites [75,76,77]. Studies have indicated that polysaccharide extracts derived from spirulina encompass a diverse mixture of maltotriose, panose, and short-chain gluco-oligosaccharides (GlcOSs). Maltotriose, classified within the category of maltodextrins (MOSs), significantly enhances the viability of *Bifidobacterium breve*, outperforming other oligosaccharides such as galacto-oligosaccharides (GOSs) and fructo-oligosaccharides (FOSs) after 24 h of fermentation [78]. During the fermentation process, MOSs facilitate the production of short-chain fatty acids (SCFAs) in the intestine while simultaneously inhibiting pathogenic bacteria [79,80]. By contrast, panose, recognized as an isomalto-oligosaccharide, encourages the growth of various *Bifidobacterium* species including *Bifidobacterium lactis*. This oligosaccharide enhances the production of butyrate and acetate during colonic fermentation [81,82]. The structural complexity of GlcOSs—including parameters such as degree of polymerization, sugar composition, stereochemistry, and various glycosidic linkages—plays a pivotal role in determining their probiotic activity, influencing the non-digestibility and selective fermentability of these oligosaccharides [77,83,84].

The elemental compositions of the microalgal biomass and polysaccharide extracts were analyzed using synchrotron X-ray fluorescence (XRF) at the Synchrotron Light Research Institute (SLRI). This advanced technique offers significant advantages in detecting inorganic mineral nutrients often present in trace amounts. Unlike traditional methods, synchrotron XRF can simultaneously identify a broad range of trace elements without the need for chemical pretreatment, providing a more accurate representation of their nutritional properties. The recent opening of Beamline 7.2 W has further expanded the potential for XRF experiments, making it possible to analyze a wide variety of elements in spirulina and its extracts. This comprehensive analysis underscores the importance of understanding the nutritional balance in this microalga, highlighting its potential as a valuable dietary component. The application of the XRF technique at BL7.2 in this study facilitated the simultaneous detection of a broad range of elements. Chemical analysis by synchrotron XRF yielded complementary information and served as a non-destructive method, enabling the examination of samples without the need for previous treatment. Our study identified elevated concentrations of sulfur, potassium, and calcium in *L. platensis* and the polysaccharide powder derived from *L. platensis*. The samples were dried, ground, pelletized, and subsequently analyzed using synchrotron XRF. The analysis also revealed essential inorganic mineral nutrients including manganese, iron, cobalt, nickel, copper, zinc, and barium in both samples, underscoring the significant advantage of the beamline in detecting inorganic mineral nutrients, which are found in considerable amounts. Elemental compositions in *Spirulina* powder samples vary in different countries including China, Bulgaria, Belgium, and the USA [85]. Elemental analysis of *Spirulina* samples by synchrotron XRF differed depending on sample origin, cultivating protocol, processing method, and environmental conditions. This technique identifies a wide variety of minor and trace elements and also evaluates inorganic minerals and nutrients, thereby providing valuable insights for enhancing nutritional knowledge in practical food applications.

**Figure 8:**
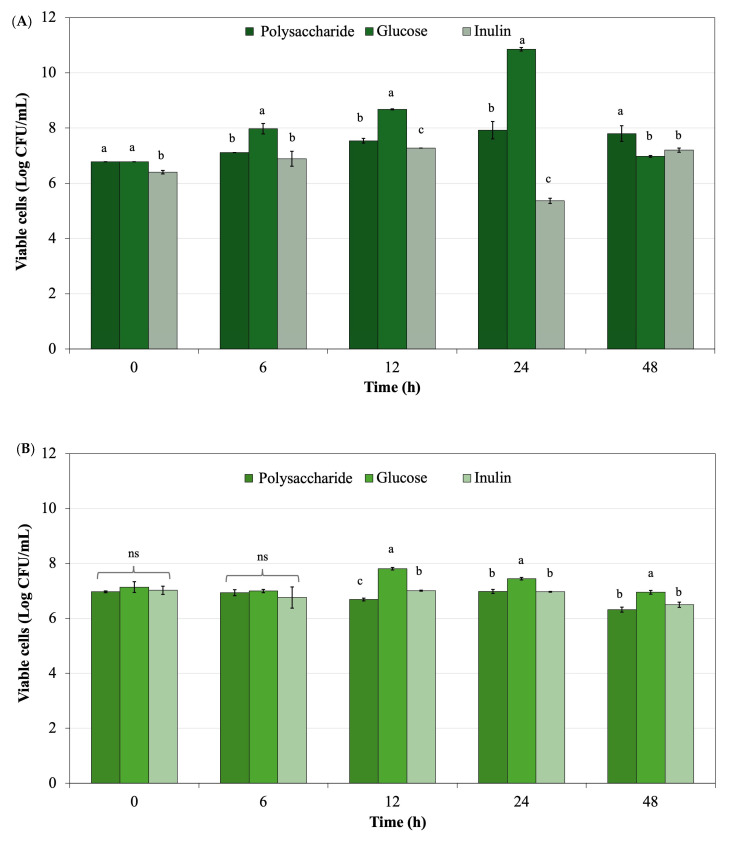
Viable cells of (**A**) *Lactobacillus rhamnosus* ATCC 53103 and (**B**) *Bifidobacterium longum* TISTR 2195 in culture broth with various supplemented ingredients, including polysaccharide powder derived from *L. platensis*, glucose, and inulin. Data with different superscripts within the same time course of fermentation are significantly different (*p* < 0.05). ns means non-significant different.

*Lactobacillus rhamnosus* and *Bifidobacterium longum* were selected for this study due to their importance as key members of the human gut microbiota and their common use in prebiotic research [86,87]. These strains have been well-studied for their probiotic properties including improving gut barrier function, modulating immune responses, and producing beneficial metabolites such as short-chain fatty acids (SCFAs) [88]. Both species effectively respond to non-digestible oligosaccharides and polysaccharides, making them ideal for evaluating the prebiotic potential of new compounds like *L. platensis*-derived polysaccharides [89]. *L. rhamnosus* has shown notable growth in response to microalgal polysaccharides [90], while *B. longum* serves as a common biomarker in functional food research due to its sensitivity to dietary fibers [91]. Thus, their inclusion allowed for a targeted assessment of the extracted polysaccharides’ selective stimulation capacity. The evaluation of polysaccharide powder from *L. platensis* to support the growth of *Lactobacillus rhamnosus* ATCC 53103 and *Bifidobacterium longum* TISTR 2195 was compared with glucose and inulin as controls. Figure 8 shows the probiotic growth with various supplemented ingredients including polysaccharide powder derived from *L. platensis*, glucose, and inulin during 48 h of culture. Viable cells of *L. rhamnosus* ATCC 53103, obtained from polysaccharide powder supplemented in the culture, were present in lower quantities than those derived from glucose, the standard medium. Viable cells from the polysaccharide powder were superior when compared with inulin, a recognized commercial prebiotic. For *B. longum* TISTR 2195, the highest viable cell count was observed in glucose culture, while polysaccharide powder derived from *L. platensis* exhibited a trend similar to inulin when supplemented in the culture.

The chemical analysis of polysaccharides derived from *L. platensis* revealed functional groups that suggested a range of health benefits. The presence of hydroxyl, carboxyl, and glycosidic linkages indicated strong antioxidant potential, capable of scavenging free radicals and reducing oxidative stress linked to chronic diseases [89]. These polysaccharides also have anti-inflammatory effects by influencing cytokine production and immune cell activation [12,92]. Their immunomodulatory properties, associated with molecular weight and structural complexity, enhance host defenses [93]. The ability to inhibit α-glucosidase also points to potential antidiabetic effects, relevant for glycemic control [94]. Overall, these bioactivities position *L. platensis* polysaccharides as promising candidates for functional foods and therapeutic agents, warranting further structural elucidation and in vivo studies to fully explore their potential.

The inhibitory effects of *Limnospira* on α-glucosidase are noteworthy. Carbohydrate metabolism relies heavily on the activity of α-amylase and α-glucosidase. Disruptions in the release and absorption of monosaccharides into the bloodstream contribute to type 2 diabetes (T2DM), which significantly affects lifestyles and has a rising mortality rate worldwide [95,96]. Targeting the inhibition of α-glucosidase is a crucial step in screening for new sources of antidiabetic compounds. Microalgae and their bioactive constituents have demonstrated considerable antidiabetic potential [97,98]. Various extracts from spirulina have been shown to suppress α-glucosidase such as water-extracted peptides (IC_50_ at 134.2 µg/mL), 80% methanol extract (IC_50_ at 9.56 mg/mL), butanol fraction (IC_50_ at 23.0 µg/mL), ethyl acetate fraction (IC_50_ at 35.7 µg/mL), and *p*-coumaric acid fraction (IC_50_ at 3.28 mg/mL) [95,99,100]. Our results indicated that polysaccharides derived from *L. platensis* can inhibit α-glucosidase activity utilizing safety and simplicity extraction technology. Future research should focus on the purification and fractionation of polysaccharides to enhance the α-glucosidase suppression effects of *Limnospira* microalgae.

In this study, Polysaccharides extracted from *L. platensis* exhibited promising bioactive properties and high yields—potentially exceeding those of other microalgae and plant sources—with significant practical applications. Their moderate α-glucosidase inhibitory activity suggests that they could serve as natural agents for managing postprandial blood glucose levels, making them valuable in diabetic supplement development [67,101]. Furthermore, their elevated phenolic and flavonoid content, along with prebiotic effects on beneficial gut bacteria like *Lactobacillus rhamnosus* and *Bifidobacterium longum*, highlights their potential in promoting gut health and metabolic balance [89,102]. Therefore, these polysaccharides demonstrate considerable promise for nutritional and therapeutic applications, with ongoing research likely to expand their scope of use.

## 5. Conclusions

The extraction of water-soluble polysaccharides from oven-dried biomass of *Limnospira platensis* has proven successful in achieving notable levels of polysaccharide content and yield, alongside total phenolics, flavonoids, and antioxidant activities. This study underscores the significance of polysaccharides derived from *L. platensis*, indicating that reducing sugars, DPPH activity, and polysaccharide content serves as a key indicator of variations influenced by extraction methods. This highlights the intricate relationship between extraction techniques and biochemical composition, emphasizing the necessity for optimized methods to fully harness these compounds. It is essential to concurrently assess the biological properties, support probiotic growth, and characterize valuable metabolites derived from microalgae. To achieve this, the use of synchrotron X-ray fluorescence (XRF) techniques at the Synchrotron Light Research Institute is recommended for elemental analysis of *Limnospira platensis* and the extracted functional polysaccharides. Future research should continue to investigate these dynamics to enhance our understanding of their potential applications. This investigation characterized the monosaccharide and oligosaccharide compositions of extracted polysaccharides but lacked comprehensive structural analyses, such as FT-IR spectroscopy, NMR, and SEC-MALS. Future research is required to utilize these techniques to better understand molecular weight distribution, glycosidic linkages, and branching structures. Measuring short-chain fatty acids (SCFAs) could validate gut fermentation outcomes and offer insights for dietary interventions to improve gut health. However, controlled studies in diverse populations are required to further clarify their roles in health and disease.

## Figures and Tables

**Table 1:** Biochemical compositions (% dry basis) of oven-dried *L. platensis* biomass.

Biochemical Composition	*L. platensis* Biomass
Ash	6.00 ± 0.20
Protein	59.93 ± 2.03
Lipid	0.94 ± 0.03
Crude fiber	16.26 ± 4.98
Carbohydrates	16.87 ± 2.72

**Table 2:** Polysaccharide concentration and extraction yield from *L. platensis* under various conditions.

Biomass/Water Ratio	Temp	Time	Cycle	Polysaccharide Concentration			Yield		
(*w*/*v*)	(°C)	(h)		(mg/mL)			(mg/g)		
1:20	70	1	1	2.11	±	0.07 ^c^	42.73	±	2.02 ^d^
			2	0.08	±	0.01 ^h^	1.56	±	0.24 ^f^
		2	1	2.48	±	0.15 ^a^	50.54	±	4.70 ^b^
			2	0.17	±	0.06 ^fg^	3.18	±	1.47 ^ef^
	90	1	1	2.33	±	0.02 ^b^	46.62	±	0.51 ^c^
			2	0.21	±	0.02 ^f^	4.03	±	0.45 ^ef^
		2	1	2.40	±	0.05 ^ab^	47.63	±	1.59 ^c^
			2	0.13	±	0.01 ^fgh^	2.66	±	0.39 ^ef^
1:40	70	1	1	1.38	±	0.04 ^d^	54.62	±	1.93 ^a^
			2	0.07	±	0.01 ^h^	2.45	±	0.88 ^ef^
		2	1	1.04	±	0.03 ^e^	41.91	±	1.92 ^d^
			2	0.12	±	0.01 ^fgh^	4.66	±	0.40 ^ef^
	90	1	1	1.34	±	0.04 ^d^	53.78	±	1.76 ^a^
			2	0.14	±	0.00 ^fgh^	5.51	±	0.26 ^e^
		2	1	1.34	±	0.01 ^d^	53.85	±	0.57 ^a^
			2	0.10	±	0.00 ^gh^	3.96	±	0.06 ^ef^

Data with different superscripts within the same column are significantly different (*p* < 0.05). Values are reported as means from triplicate experiments ± standard deviation (SD).

**Table 3:** The values of TPC, TFC, and reducing sugars of polysaccharides extracted from *L. platensis* under various conditions.

Biomass/Water Ratio	Temp	Time	Cycle	TPC			TFC			Reducing Sugar		
(*w*/*v*)	(°C)	(h)		(mg GAE/g)			(mg QE/g)			(mg/g)		
1:20	70	1	1	3.19	±	0.03 ^d^	0.61	±	0.00 ^e^	4.77	±	0.31 ^a^
			2	0.40	±	0.01 ^f^	0.18	±	0.01 ^h^	0.00	±	0.00 ^f^
		2	1	3.24	±	0.06 ^d^	0.57	±	0.01 ^f^	4.58	±	0.02 ^ab^
			2	0.39	±	0.01 ^f^	0.19	±	0.00 ^h^	0.00	±	0.00 ^f^
	90	1	1	3.09	±	0.04 ^e^	0.64	±	0.04 ^d^	4.47	±	0.04 ^b^
			2	0.38	±	0.01 ^f^	0.22	±	0.01 ^g^	0.00	±	0.00 ^f^
		2	1	3.20	±	0.02 ^d^	0.74	±	0.02 ^b^	4.67	±	0.04 ^ab^
			2	0.42	±	0.01 ^f^	0.22	±	0.00 ^g^	0.00	±	0.00 ^f^
1:40	70	1	1	3.40	±	0.10 ^c^	0.63	±	0.00 ^de^	1.35	±	0.07 ^c^
			2	0.00	±	0.00 ^g^	0.18	±	0.01 ^h^	0.00	±	0.00 ^f^
		2	1	3.53	±	0.01 ^b^	0.68	±	0.02 ^c^	0.74	±	0.19 ^d^
			2	0.00	±	0.00 ^g^	0.17	±	0.02 ^h^	0.00	±	0.00 ^f^
	90	1	1	3.38	±	0.09 ^c^	0.75	±	0.01 ^b^	0.50	±	0.37 ^e^
			2	0.00	±	0.00 ^g^	0.20	±	0.01 ^h^	0.00	±	0.00 ^f^
		2	1	3.66	±	0.05 ^a^	0.90	±	0.03 ^a^	0.84	±	0.13 ^d^
			2	0.00	±	0.00 ^g^	0.20	±	0.00 ^h^	0.00	±	0.00 ^f^

Data with different superscripts within the same column are significantly different (*p* < 0.05). Values are reported as means from triplicate experiments ± standard deviation (SD).

**Table 4:** Antioxidant properties of polysaccharides extracted from *L. platensis* under various conditions.

Biomass/Water Ratio	Temp	Time	Cycle	DPPH			ABTS			FRAP		
(*w*/*v*)	(°C)	(h)		(mg AAE/g)			(mg AAE/g)			(mg AAE/g)		
1:20	70	1	1	0.29	±	0.01 ^f^	1.51	±	0.01 ^de^	1.10	±	0.02 ^g^
			2	0.14	±	0.00 ^gh^	0.68	±	0.00 ^g^	0.47	±	0.01 ^l^
		2	1	0.31	±	0.00 ^e^	1.49	±	0.01 ^e^	1.18	±	0.01 ^f^
			2	0.15	±	0.00 ^g^	0.82	±	0.03 ^f^	0.46	±	0.01 ^l^
	90	1	1	0.61	±	0.00 ^a^	1.56	±	0.03 ^d^	1.57	±	0.00 ^d^
			2	0.14	±	0.00 ^gh^	0.62	±	0.06 ^h^	0.64	±	0.01 ^j^
		2	1	0.56	±	0.01 ^c^	1.52	±	0.01 ^de^	1.67	±	0.01 ^c^
			2	0.14	±	0.00 ^gh^	0.60	±	0.01 ^h^	0.62	±	0.04 ^j^
1:40	70	1	1	0.58	±	0.01 ^b^	2.27	±	0.02 ^a^	1.46	±	0.02 ^e^
			2	0.12	±	0.00 ^i^	0.73	±	0.02 ^g^	0.58	±	0.02 ^k^
		2	1	0.30	±	0.01 ^ef^	2.26	±	0.02 ^ab^	1.42	±	0.06 ^e^
			2	0.13	±	0.00 ^hi^	0.61	±	0.06 ^h^	0.56	±	0.01 ^k^
	90	1	1	0.54	±	0.00 ^d^	2.08	±	0.03 ^c^	1.86	±	0.03 ^b^
			2	0.14	±	0.01 ^gh^	0.48	±	0.02 ^j^	0.71	±	0.01 ^i^
		2	1	0.55	±	0.03 ^cd^	2.22	±	0.05 ^b^	1.91	±	0.05 ^a^
			2	0.15	±	0.00 ^g^	0.53	±	0.01 ^i^	0.78	±	0.02 ^h^

Data with different superscripts within the same column are significantly different (*p* < 0.05). Values are reported as means from triplicate experiments ± standard deviation (SD).

**Table 5:** Compositions of monosaccharides (mg/g) in polysaccharide powder and acid-hydrolyzed polysaccharide powder obtained from *L. platensis*.

Composition	Polysaccharide Powder	Acid-Hydrolyzed Polysaccharide Powder
Rhamnose	n.d.	21.99 ± 0.35 ^b^
Arabinose	n.d.	0.44 ± 0.02 ^e^
Galactose	n.d.	16.14 ± 0.49 ^c^
Glucose	9.67 ± 0.40 ^a^	63.27 ± 5.11 ^a^
Xylose	n.d.	6.51 ± 0.21 ^d^
Fructose	0.40 ± 0.01 ^b^	0.43 ± 0.02 ^e^

Values are presented as mean ± standard deviation (SD), where “n.d.” denotes not detected. Data with different superscripts within the same column are significantly different (*p* < 0.05).

**Table 6:** Weight percentages from X-ray fluorescence (XRF) analysis.

Element	Weight (%)	
	*L. platensis* Biomass	Polysaccharide Powder
Sulfur (S)	50.12 ± 0.05	25.41 ± 0.16
Chlorine (Cl)	0.89 ± 0.03	3.10 ± 0.04
Potassium (K)	25.17 ± 0.43	55.99 ± 0.16
Calcium (Ca)	20.63 ± 0.44	15.12 ± 0.08
Titanium (Ti)	0.05 ± 0.00	n.d.
Chromium (Cr)	0.01 ± 0.00	n.d.
Manganese (Mn)	0.14 ± 0.00	0.08 ± 0.00
Iron (Fe)	2.62 ± 0.05	0.26 ± 0.00
Cobalt (Co)	0.01 ± 0.00	0.001 ± 0.00
Nickel (Ni)	0.01 ± 0.00	0.0005 ± 0.00
Copper (Cu)	0.05 ± 0.00	0.01 ± 0.00
Zinc (Zn)	0.27 ± 0.01	0.01 ± 0.00
Barium (Ba)	0.048 ± 0.00	0.03 ± 0.00

Values are presented as mean ± standard deviation (SD), where “n.d.” denotes not detected.

**Table 7:** Evaluation of α-glucosidase inhibition activities in biomass and polysaccharide powder derived from *L. platensis*.

Treatment	IC_50_ (mg/mL)
*L. platensis* biomass	NA *
Polysaccharide powder	33.01 ± 0.89 ^a^
Acarbose	3.41 ± 0.44 ^b^

IC_50_ means the concentration of the sample that inhibits α-glucosidase activity by 50%. Values expressed as mean ± standard deviation (SD). * Means IC_50_ value is more than 50 mg/mL. Data with different superscripts are significantly different (*p* < 0.05).

**Table 8:** Monosaccharide composition from *Limnospira*.

Strain	Source	Ara	Fuc	Fru	Gal	Glc	Man	Rha	Xyl	Ref.
*L. platensis* SAG 21.99	Germany	n.d.	n.d.	n.d.	0.7	24.6	0.5	n.d.	n.d.	[7]
*L. platensis* F&M-C256	Italy	<10.0	<10.0	n.d.	11.0	18.0	n.d.	55.0	<10.0	[74]
*L. platensis* F&M-C260	Italy	<10.0	<10.0	n.d.	11.0	30.0	<10.0	21.0	10.0	[74]
*L. platensis* (FACHB: GY-D18)	China	0.6	3.3	n.d.	n.d.	83.6	n.d.	4.4	1.1	[71]
SPO-1 from *L. platensis*	China	n.d.	n.d.	n.d.	5.4	94.6	n.d.	n.d.	n.d.	[30]
*Polysaccharides derived from L. platensis*	Thailand	0.4	n.d.	0.4	16.1	63.3	n.d.	22.0	6.5	This study

Ara—arabinose, Fuc—fucose, Fru—fructose, Gal—galactose, Glc—glucose, Man—mannose, Rha—rhamnose, Xyl—xylose; SPO-1 means *Limnospira* oligosaccharide (DP = 4, MW = 650.2 kDa); n.d. means not detected.

## Data Availability

The original contributions presented in this study are included in the article.

## Abbreviations

The following abbreviations are used in this manuscript:

TPC	Total phenolic content
TFC	Total flavonoid content
R. sugars	Reducing sugar
DPPH	2,2-diphenyl-1-picrylhydrazyl
ABTS	2,2-azino-bis (3-ethylbenzothiazoline-6-sulfonic acid)
FRAP	Ferric ion reducing antioxidant power
GAE	Gallic acid equivalent
AAE	Ascorbic acid equivalent
PS	Polysaccharide
XRF	X-ray fluorescence
